# The Duty Incumbent upon Dentists to Attend Associations

**Published:** 1871-02

**Authors:** Arthur S. Ford


### ARTICLE IV.

### *The Duty incumbent upon Dentists to attend Associations.*

By Arthur S. Ford, D. D. S.

As the time rapidly approaches for the annual session of the Southern Dental Association, I think it not inappropriate to impress upon the minds of my professional brethren the duty that they owe not only to themselves, but also to their profession and their patients, to embrace every opportunity to perfect themselves as operators, and more especially at this present time, when the whole scientific world is on the "qui vive" for improvements, and when, as I fondly believe, our own profession is keeping pace with the "march of intellect" in the known professions, and is making giant strides towards perfection, by substituting improved modes of treatment and operating, together with new instruments and appliances.

There is, in my humble opinion, nowhere, such an opportunity offered to the practitioner for advancement towards proficiency, by the interchange of modes of practice and by seeing and learning all the latest improvements in operating, in instruments and material, as that afforded by attending Dental Associations and Societies. Such is admitted to be a fact by all progressive dentists, and if true with regard to local and State organizations, how much more so must it be, when the accumulated learning and experience of the whole country is convened in a general Association.

I would therefore urge upon my professional brethren, North, South, East and West, to forego all but providential, considerations, and let us meet in Charleston S. C., for mutual improvement on the the second Wednesday in April next.

Let not any ignore the capabilities of others to instruct, or too modestly underate their own abilities to impart knowledge. We are all susceptible of improvement, and few there are that cannot advance some original idea that may be of advantage to the many.

Therefore let us lay aside all private prejudices, and sacrifice all personal benefits for the advancement of our profession and the good of mankind, and let us each in the approaching sessions of the Southern Dental Association, strive to learn or teach according to our abilities, and labor assiduously to elevate the standard of our honored profession not only in America but throughout the world.

---

